# Successful eradication of newly acquired MRSA in six of seven patients with cystic fibrosis applying a short-term local and systemic antibiotic scheme

**DOI:** 10.1186/s12890-018-0588-6

**Published:** 2018-01-25

**Authors:** Alexander Kiefer, Christian Bogdan, Volker O. Melichar

**Affiliations:** 1 Department of Pediatrics and Adolescent Medicine, Clinic for Cystic Fibrosis, Universitätsklinikum Erlangen, Loschgestraße 15, 91054 Erlangen, Germany; 2 Microbiology Institute – Clinical Microbiology, Immunology and Hygiene, Universitätsklinikum Erlangen, Friedrich-Alexander-Universität (FAU) Erlangen-Nürnberg, Wasserturmstraße 3/5, 91054 Erlangen, Germany

**Keywords:** Cystic fibrosis, Methicillin- resistant *Staphylococcus aureus* (MRSA), Eradication

## Abstract

**Background:**

In individuals with cystic fibrosis (CF), colonization with methicillin-resistant *Staphylococcus aureus* (MRSA) was reported to be associated with a deterioration of pulmonary disease as reflected by an accelerated decline in lung function. Thus, an early eradication of MRSA could be beneficial in these patients. Here, we report on an intensified MRSA eradication protocol.

**Methods:**

Since 2012 a protocol for the eradication of newly acquired MRSA has been used in our CF Clinic, combining oral rifampicin and fusidic acid, inhaled vancomycin, nasal mupirocin, local antiseptic treatment and hygienic directives all of which are applied for only 7 days during an inpatient hospital stay.

**Results:**

Since 2012 seven patients (3 male, 4 female; age range 4 to 30 years) newly acquired MRSA. In 6 of the 7 patients (86%) successful eradication of MRSA was achieved upon first treatment using the protocol described above. In one patient a second course of treatment was performed which, however, also failed to eliminate the colonizing MRSA.

**Conclusions:**

Our protocol led to an eradication rate of 86%. The impact of each individual component of the protocol remains to be determined.

## Background

The prevalence of methicillin-resistant *Staphylococcus aureus* (MRSA) colonization in patients with cystic fibrosis (CF) has increased over the past years. The carrier rate significantly varies between regions, ranging from 3,4% in some CF populations in the United Kingdom [1] as high as 30% in the United States [2–5]. The chronic colonization with MRSA seems to be associated with a worsening of the pulmonary disease, with an accelerated decline in lung function and/or a prolonged recovery period after clinical exacerbations [1–3, 6, 7]. Several protocols for an early eradication of MRSA have been reported to prevent negative consequences that might result from chronic MRSA colonization [1, 2, 8]. To the best of our knowledge, studies systematically comparing these protocols are lacking in the literature, as recently also highlighted by a Cochrane review [1, 9, 10]. The aim of this retrospective study was to evaluate the efficacy of our protocol which consists of oral rifampicin and fusidic acid, inhaled vancomycin, nasal mupirocin, and hygienic directives over 7 days. This protocol has been used in our CF center since 2012.

## Methods

In 2012 we established a standardized eradication protocol for newly colonized patients with CF. This protocol consists of oral rifampicin (7.5–10 mg/kg, maximum 300 mg twice daily) and oral fusidic acid (15 mg/kg maximum 500 mg three times daily). The combination of these two antibiotics seems to be effective against MRSA in CF patients [1, 8, 11]. In addition, an inhalation therapy with vancomycin (4 mg/kg, maximum 250 mg, dissolved in 4 ml sodium chloride 0,9% twice daily) is applied. None of the MRSA strains isolated from the CF patients was resistant to the above-mentioned antibiotics. Supplementary hygienic measurements including the application of mupirocin ointment and the use of disinfectants were performed as stated in Table 1. All these measures were performed under inpatient hospital conditions and contact precautions over 7 days; the inhalation therapy was supervised by a trained physiotherapist. Before starting the treatment, every family member and the pets living in the same household were also tested for MRSA. These test remained negative in each of our patients.

**Table 1:** Local measures

• Hand sanitation (Alcohol-based: Desderman ®, Sterillium®) as often as possible and reasonable	
• Application of mupirocin into the nasal atrium: three times daily	
• Disinfecting full body wash including the hair using antiseptic solutions (octenidin, polihexanide): once daily	
• Rinse of the oropharynx with antiseptic: chlorhexidine, hexetidine or octenidin: three times daily	
• Replacement of toothbrush or soaking in chlorhexidine: after use	
• Change of underwear, clothes and bedding and disinfecting laundering: once daily	
• Disinfection of all surfaces in the room with glucoprotamin 0,5%: once daily	

Since 2012 seven patients (3 male, 4 female) were newly colonized with MRSA and all were treated according to the above-mentioned protocol. The age of the patients ranged from 4 to 30 years, with a median age of 15 years. Prior to the start of the treatment each patient or its parents or legal guardian, if the patient was under 18 years old, gave oral informed consent to undergo this therapy scheme. None of the patients who were offered eradication refused to undergo the treatment, and all patients completed the treatment. New colonization with MRSA was defined by one positive culture for MRSA using either sputum analysis or an oropharyngeal swab. The cultures were performed as part of our routine monitoring, which in our CF center is carried out at least every 3 months. One patient acquired MRSA again 9 months after the first successful eradication and therefore underwent eradication for a second time. The age of the patients ranged from 4 to 30 years, with a median age of 15 years. The MRSA was either detected in sputum cultures (4 patients) or in deep oropharyngeal swabs (3 patients). Clinical data of the patients such as age, sex, and FEV1 determined at the time of MRSA detection are summarized in Table 2.

**Table 2:** Clinical data of the patients (at the time of the first MRSA detection)

Patient no.	Genotype	FEV1% predicted	bacterial colonization (other than MRSA)
Patient 1	F508del/2721del11	76,4%	*Pseudomonas aeruginosa*
Patient 2	F508del/F508del	31,3%	*Pseudomonas aeruginosa*
Patient 3	F508del/Arg334Trp	72,0%	MSSA^a^
Patient 4	F508del/F508del	80,1%	none
Patient 5	F508del/del17	51,5%	MSSA, *Burkholderia cepacia*
Patient 6	F508del/F508del	Not possible	none
Patient 7	F508del/F508del	76,3%	MSSA, *Pseudomonas aeruginosa*

^a^MSSA, methicillin-susceptible *Staphylococcus aureus*

Bacterial cultures of sputum and/or of oropharyngeal swabs were obtained on day 4, 5 and 6 after the eradication attempt. If all the tests remained negative for MRSA, a successful eradication was assumed. The patients were then followed up every 3 months. In addition to the microbiological analyses a lung function test was performed if the patient was able to do it. The entire lung function tests were executed in a Master-ScreenTM Body plethysmograph (Jaeger) by a specially trained nurse.

## Results

Successful eradication was achieved in 6 of 7 patients (86%). In one patient the first eradication attempt was unsuccessful and was repeated using the same protocol, which, however, also failed. In another patient MRSA was detected again after 9 months, but was again successfully eradicated. As the MRSA isolates were not subjected to molecular typing, we cannot tell whether the patient had a recurrent colonization with the same MRSA strain or whether he acquired a new MRSA strain.

Due to the retrospective design of our study, the follow-up period of the patients varied between 7 and 38 months (median 16 months) (see Table 3 for more details).

**Table 3:** Rate of change in FEV1% predicted and time of follow up

Patient no	Change in FEV1% predicted per year	Time of follow up (months)
1	- 0.82	59
2	−1.26	57
3	+ 0.86	28
5	- 8.49	24
7	−0.7	17

**Fig. 1:**
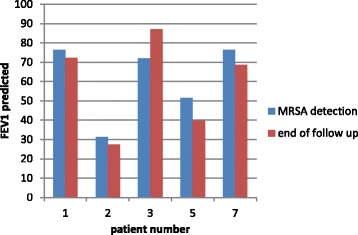
FEV1% predicted in patients who underwent successful eradication of MRSA. In patient 4 MRSA was not eradicated; patient 6 was too young to perform lung function test

One of the patients (number 6) that successfully underwent the MRSA eradication protocol, was too young to perform a standard lung function test. In the other patients the rate of change in FEV1% predicted varied between a decline of 8.49 per year (patient with a co-colonization with *Burkholderia cepacia*) and an improvement of 0.86 points. The absolute change in FEV1% predicted between the time of initial MRSA detection and the end of the follow up period is shown in Fig. 1. The details of changes in FEV1% predicted per year are shown in Table 3.

## Discussion

The impact of a chronic colonization with MRSA in individuals with cystic fibrosis is still a matter of debate. Some studies documented a worsening of pulmonary disease and accelerated loss of lung function in CF patients colonized with MRSA [1–3, 6, 7]. On the other hand, some studies suggest that acquisition of MRSA is not associated with an accelerated loss of lung function [12]. Although a positive effect on pulmonary disease and lung function parameters is not proven yet, there are other issues that have to be considered. Because of the contact precautions, which are necessary for patients with chronic MRSA colonization, they are often unable to receive important therapeutic measures which are part of the routine treatment for CF patients in Germany: In particular the outpatient physiotherapy, which is performed once a week by a specially trained physiotherapist and the rehabilitations are frequently withheld from the patients. This might be different in other countries, but in our opinion this is an important reason to eradicate MRSA. Several CF centers therefore aim at an early eradication of MRSA immediately after its first detection. The protocols used for the eradication attempts are difficult to compare as they differ in terms of the type, administration route and duration of antibiotic therapy. The success rate of eradication observed with our protocol (86%) compares well to other published schemes, in which the success rates ranged from 55% (Solis et al.) [13] to 94% (Macfarlane et al.) [8]. However, the published studies are not directly comparable, as there are many differences especially with respect to the applied antibiotics, the duration of the therapy, and the follow-up period (see Table 4 for more details). A notable advantage of our eradication protocol is its short duration of 7 days. This might not only lead to a better compliance of the patients, but should also have a positive impact with respect to side effects and the development of antibiotic resistance. The fact that the eradication scheme was performed in an inpatient hospital setting is likely to be an important factor for the high success rate, because adherence to the therapy and especially the implementation of the local measures will be higher under direct supervision than at home. In our opinion eradication according to this protocol at home would lead to a significantly lower success rate. However, as there was no control group who underwent the eradication protocol at home, this remains to be formally demonstrated. In Germany, the inpatient hospital stays are covered by the health insurances. Therefore, we have to admit that this protocol may not be feasible in other countries. Our protocol consists of three parts (local measures, oral rifampicin and fusidic acid and inhaled vancomycin). Unfortunately, we are unable to make a statement on the impact of each individual measurement on the overall success of our protocol. In particular the value of the inhaled vancomycin remains unclear. However, treatment periods longer than 7 days are probably not more successful, as we observed comparable clearance rates. In summary our protocol was well tolerated an accepted by the patients and provided an effective way to eradicate MRSA. The fact that none of the patients who have been offered the eradication procedure refused to undergo the treatment or interrupted the protocol emphasizes the compliance of the patients and the acceptance of the procedure.

**Table 4:** Overview on studies that evaluated different antibiotic protocols for the eradication of MRSA after its first detection

Author	Patients treated	Age of patients (years)	Treatment protocol	Eradication success (%)
Solis et al. [13]	12	median 9.8 (0,6–17)	5 days topic/inhaled vancomycin	58
Macfarlane et al. [8]	17	mean 12.3 (1.8–16.5)	1. oral rifampicin and fusidic acid over 5 days	47%
			2. If still positive: oral rifampicin and fusidic acid over additional 5 days	71%
			3. If still positive: intravenous teicoplanin	94%
Hall et al. [1]	29	mean 30 (17–62)	either single or dual oral antibiotic therapy, until negative sputum, minimum 2 weeks, maximum 8 weeks	single therapy 50% dual therapy 85%
Kappler et al. [17]	37	mean 15.3 (0.6–36.9)	3 weeks dual intravenous + 6 weeks dual oral antibiotics + inhaled vancomycin if still positive 6 weeks inhaled vancomycin	84
Muhlebach et al. *Treatment* [16]	22	mean 12.3 (SD 6.6)	2 weeks dual oral antibiotics (rifampicin and trimethoprim/sulfamethoxazole or rifampicin an minocycline)	82
Muhlebach et al. *Observational control* [16]	19	mean 10.5 (SD 5.5)	none	26
this study	7	Median 15 (4–30)	Oral rifampicin and fusidic acid + inhaled vancomycin over 7 days	86

Major limitations of our study are the low number of cases and the retrospective design. In addition, we used different specimens (sputum and/or deep oropharyngeal swab) to detect MRSA. This was necessary because some patients were unable to produce sputum. Previous publications suggested that cultures of oropharyngeal swabs are not reliable to predict the presence of bacterial pathogens in the lower airways of cystic fibrosis patients [14, 15]. However, given the fact that we collected three independent specimens for culture after MRSA eradication and that the patients remained negative for a median period of 16 months, we believe that the risk of a false negative result is minimal. Moreover, the heterogeneity of the patients with respect to age, sex and coinfection or co-colonization with other bacteria such as *Pseudomonas aeruginosa* and *Burkholderia cepacia* made it impossible to demonstrate a positive clinical effect of MRSA eradication in our patients. Furthermore, we were unable to compare our results to a control group of patients that were not offered eradication. A recent publication showed a spontaneous elimination of MRSA of only 26% of the affected patients [16]. In our opinion this emphasizes the importance of MRSA eradication, which, in our opinion will be beneficial for the patients in the long run.

As mentioned above and confirmed recently by the Cochrane Collaboration [9, 10], a comparison of all published eradication schemes is not possible. Further and prospective studies comparing several eradication protocols for MRSA would be useful.

## Conclusion

Our short term antibiotic protocol led to the eradication of newly acquired MRSA at a rate of 86%, which compares well to other published schemes. The impact of each individual component of the protocol remains to be determined.

## Abbreviations

CF: Cystic fibrosis
FEV1: Forced expiratory volume in one second
MRSA: Methicillin-resistant *Staphylococcus aureus*
MSSA: Methicillin-susceptible *Staphylococcus aureus*
